# Secretome of Mesenchymal Stromal Cells Prevents Myofibroblasts Differentiation by Transferring Fibrosis-Associated microRNAs within Extracellular Vesicles

**DOI:** 10.3390/cells9051272

**Published:** 2020-05-20

**Authors:** Nataliya Basalova, Georgy Sagaradze, Mikhail Arbatskiy, Evgeniy Evtushenko, Konstantin Kulebyakin, Olga Grigorieva, Zhanna Akopyan, Natalia Kalinina, Anastasia Efimenko

**Affiliations:** 1 Institute for Regenerative Medicine, Medical Research and Educational Centre, Lomonosov Moscow State University, 119192 Moscow, Russia; natalia_ba@mail.ru (N.B.); gsagaradze@mc.msu.ru (G.S.); go.grigorievaolga@gmail.com (O.G.); zhanna.fbm@gmail.com (Z.A.); 2 Faculty of Medicine, Lomonosov Moscow State University, 119192 Moscow, Russia; algenubi81@mail.ru (M.A.); konstantin-kuleb@mail.ru (K.K.); n_i_kalinina@mail.ru (N.K.); 3 Faculty of Chemistry, Lomonosov Moscow State University, 119991 Moscow, Russia; evtushenko@enzyme.chem.msu.ru

**Keywords:** mesenchymal stem/stromal cells, fibrosis, myofibroblasts, secretome, extracellular vesicles, microRNA

## Abstract

Fibroblasts differentiation into myofibroblasts is a central event of tissue fibrosis. Multipotent mesenchymal stromal cells (MSCs) secretome can interfere with fibrosis development; despite precise underlying mechanisms remain unclear. In this study, we tested the hypothesis that MSC secretome can affect fibroblast’ differentiation into myofibroblasts by delivering regulatory RNAs, including microRNAs to these cells. Using the model of transforming growth factor-beta (TGFbeta)-induced fibroblast differentiation into myofibroblasts, we tested the activity of human MSC secretome components, specifically extracellular vesicles (MSC-EV). We showed that MSC-EV down-regulated secretion of extracellular matrix proteins by fibroblasts as well as suppressed their contractility resulting in prevention as well as reversion of fibroblasts differentiation to myofibroblasts. High-throughput sequencing of RNAs extracted from MSC-EV has revealed many fibrosis-associated microRNAs. Fibroblast treatment with MSC-EV led to direct transfer of microRNAs, which resulted in the elevation of most prominent fibrosis-associated microRNAs, including microRNA-21 and microRNA-29c. Using MSC-EV transfection by antagomirs to these microRNAs we demonstrated their involvement in the suppression of fibroblast differentiation in our model. Taken together, MSC secretome can suppress fibrosis by prevention of fibroblast differentiation into myofibroblasts as well as induce de-differentiation of the latter by direct transfer of specific microRNAs.

## 1. Introduction

Tissue repair after injury can result in complete restoration of tissue structure as well as replacement of a part of the parenchyma with components of the stroma. Many pathological conditions can increase the stromal component and lead to fibrosis, which primarily occurs as a result of excessive deposition of extracellular matrix (ECM) proteins, such as collagen and fibronectin, in the tissues. The main source of ECM are myofibroblasts and therefore their differentiation from multiple sources, including fibroblasts and endothelial cells is the central event of fibrosis development [1].

Multipotent mesenchymal stem/stromal cells (MSCs) or their conditioned medium (MSC-CM) suppressed fibrosis development in various in vivo models, such as kidney failure, liver fibrosis, and idiopathic pulmonary fibrosis [2,3,4]. However, the mechanisms of these effects are still poorly understood. Analysis of MSC secretome has revealed multiple ECM-remodeling enzymes, including matrix metalloproteinases (MMP2 and MMP9) and their inhibitors (TIMP-1 and TIMP-2) as well as fibrosis-related cytokines such as IL-10 and prostaglandin-E2 and growth factors like vascular endothelial growth factor (VEGF) and hepatocyte growth factor, (HGF) [5,6,7,8,9]. More recently the significant input of regulatory RNA in many effects of MSCs secretome, including stimulation of angiogenesis and wound healing, was demonstrated. Regulatory or non-coding RNAs, including microRNA (miRs), piwiRNA, lncRNA, Y-RNA and many others, together represent the largest part of mammalian transcriptome, which is not translated into proteins but participates in many regulatory steps of chromatin compactization, transcription, and translation (for recent reviews see [10]). Among those, the biological role of miRs is the most understood. These 19–25 nucleotides RNAs decrease the expression of messenger RNAs that contain complementary sequences. Selected miRs are secreted as complexes with Argonaute proteins or Nucleophosmin-1 as well as within extracellular vesicles (MSC-EV). Currently the physiological roles of various MSC secretome components are being intensively explored. MSC-EV could transfer receptors, growth factors, lipids, and different types of RNA, including regulatory miRs, from MSCs to target cells, allowing simultaneous transmission of different molecules at high concentrations over long distances [11,12,13].

In this study, we explored the role of factors secreted by MSCs in fibrogenesis using a model of transforming growth factor-beta (TGFbeta)-induced differentiation of fibroblasts into myofibroblasts as a crucial process in the pathogenesis of fibrosis. We demonstrated the specific ability of MSC-CM fractions, preferentially MSC-EV, to prevent the differentiation of fibroblasts into myofibroblasts and even induce reverse differentiation back into fibroblasts. Using this established in vitro model, we demonstrated a direct transfer of fibrosis-associated miRs within MSC-EV to fibroblasts and involvement of miR-21 and miR-29c in MSC-EV mediated antifibrotic effects.

## 2. Materials and Methods

### 2.1. Cell Culture

ASC52telo, hTERT immortalized adipose derived mesenchymal stem cells (ATCC^®^ SCRC-4000™) were maintained in the medium supporting the growth of undifferentiated mesenchymal progenitor cells (Advance Stem Cell Basal Medium, HyClone, Logan, UT, USA) containing 10% of a growth factor supplement (Advance Stem Cell Growth Supplement, HyClone), 100 U/mL of penicillin/streptomycin (Gibco, Thermo Fisher Scientific, Carlsbad, CA, USA), medium was changed every 2–3 days. Cells were passaged at ≈80% confluency. All experiments were performed with cells from 15 to 25 passages.

MSCs derived from adipose tissue of healthy donors (*n* = 3) were obtained from the biobank of the Institute for Regenerative Medicine, Lomonosov MSU, collection ID: MSU_MSC_AD (https://human.depo.msu.ru) and cultured in the medium supporting the growth of undifferentiated mesenchymal progenitor cells (Advance Stem Cell Basal Medium, HyClone) containing 10% of a growth factor supplement (Advance Stem Cell Growth Supplement, HyClone), 100 U/mL of penicillin/streptomycin (Gibco). Medium was changed every 3–4 days. All experiments were performed with cells within 5 passages. All procedures performed with tissue samples from patients were in accordance with the Declaration of Helsinki and approved by the Ethic Committee of Lomonosov Moscow State University (IRB00010587), protocol #4 (2018).

Human dermal fibroblasts (HDF) were obtained from the biobank of the Institute for Regenerative Medicine, Lomonosov MSU, collection ID: MSU_FB (https://human.depo.msu.ru) and cultured in DMEM with low glucose supplemented with 10% FBS and 1% penicillin-streptomycin (all from Gibco). Medium was changed every 3–4 days. All experiments were performed with cells within 12 passages.

To induce differentiation of HDF to myofibroblasts, cells were seeded in 12-well plates in concentration 4 × 10^5^ cells per well, grown for 1 day and serum deprived overnight. Then, the fresh serum-free culture medium was applied together with 5 ng/mL TGFbeta (R&D, Minneapolis, MN, USA) and one of MSC-CM fractions (800 μL per well). DMEM LG without FBS was utilized as a negative control; DMEM LG without FBS with 5 ng/mL TGFbeta was used as positive control. Cells were placed into CO^2^-incubator at 37 °C and assayed after 4 days.

To induce redifferentiation of myofibroblasts back to fibroblasts, cells were seeded in 12-well plates in concentration 4 × 10^5^ cells per well, grown for 1 day and serum deprived overnight. Then, the fresh serum-free culture medium was applied together with 5 ng/mL TGFbeta (R&D) for 4 days. After that cells were washed with Hanks’ buffer solution (Paneco) and then one of MSC-CM fractions or DMEM LG without FBS were added (800 μL per well). Cells were placed into CO2-incubator at 37 °C and assayed after 4 days.

### 2.2. Conditioned Medium Harvesting and Fractioning

For producing MSC-CM used for cell treatment, MSC were seeded in 10 cm dishes (~2 × 10^3^ cells/cm^2^). At 90–100% confluence, cells were washed with Hanks’ buffer solution (Paneco) for three times. Then dishes were incubated for 48 h with 10 mL DMEM LG at 37 °C, 5% CO2. DMEM LG with no cells was used as a negative control (DMEM). After 48 h, medium was removed and centrifuged for 10 min at 300× *g* to remove cell debris. MSC-CM was concentrated on Minim™ II Tangential Flow Filtration System, 10 kDa filters (Pall Corporation, Port Washington, NY, USA), then transferred to an Amicon filter (1000 kDa, Sigma, Milan, Italy) and centrifuged to separate extracellular vesicles (MSC-EV) fraction from the EV-depleted fraction of MSC-CM (MSC-SF). Both fractions were concentrated up to 5 times. All samples were stored at −80 °C. Average cell count following removal of MSC-CM for concentration was 6.8 × 10^5^ cells.

Characterization of MSC-EV was performed by nanoparticle tracking analysis (NTA), transmission electron microscopy (TEM), and Western blot analysis (see below). Since EVs are not the only particles might be counted by NTA even in serum-free CM, two types of samples were analyzed by NTA: Aliquots of MSC-CM after 30 min of incubation with fresh serum-free media (DMEM LG) and after subsequent 48 h of incubation. Sample after only 30 min of incubation with cell served as a control of adventitious particles in medium prior to EVs release. Both non-concentrated and concentrated samples of MSC-CM were analyzed.

### 2.3. Nanoparticle Tracking Analysis

Characterization of EVs by NTA was made with Nanosight LM10 (Nanosight Ltd., Salisbury, UK), equipped with 405 nm, 65 mW laser unit with passive temperature readout and high sensitivity Andor Luca camera of EMCCD type. All measurements were performed according to the recommendations of the ASTM E2834-12 standard [14] using the protocol [15], optimized for EVs under this particular Nanosight instrument configuration. Due to slight fluorescent background of CM, minor adjustments of lower and higher thresholds were made (910 and 10,920 instead of 715 and 10,725 respectively).

### 2.4. TEM Imaging

TEM imaging was conducted for MSC-EV loaded on carbon-coated grids, subjected to negative glow-discharging in an air atmosphere and stained on drops (40 μL) of 1% uranyl acetate solution twice for 30 s each time. Grids were imaged at 80 kV with a JEOL JEM-1011 TEM with digital camera ORIUS SC1000W.

### 2.5. Immunofluorescent Analysis

HDF grown on uncoated glass coverslips were fixed with 4% paraformaldehyde in PBS for 15 min at RT and permeabilized with 0.1% Triton X-100 for 10 min at RT. After blocking nonspecific binding with 10% normal goat serum on 1% BSA (Abcam, Cambridge, UK), cells were incubated with antibody to a-SMA (ab32575; Abcam), rabbit IgG (NSC-2025; Santa Cruz Biotechnology, Santa Cruz, CA, USA), vinculin (V4139; Sigma) or Alexa 594-phalloidin (А12381; Molecular probe, Eugene, OR, USA) overnight at 4 °C. Antibody detection was performed using secondary antibodies conjugated with Alexa 488 (A11037; Invitrogen, Life Technologies, Grand Island, NY, USA) for 1 h at RT in the dark. The nuclei were counterstained with DAPI (D9542; Sigma,). Coverslips were mounted in AquaPolyMount (Polysciences Inc., Warrington, PA, USA). Images were captured using a Leica DM600B with a Leica DFC 420C camera (Leica Microsystems GmbH, Mannheim, Germany) and processed by FIJI software (GitHub Inc., Microsoft, Redmond, WA, USA).

### 2.6. Collagen Gel Contraction Assay

Collagen gel contraction assay was performed as described previously [16]. Briefly, cells were trypsinized and washed with PBS. 8 × 10^4^ cells per well in investigated samples (400 μL), containing 5 ng/mL of TGFbeta (R&D) were added to the collagen (3 mg/mL in 0.1% acetic acid, Imtek, Moscow, Russia). The matrix was allowed to polymerize at room temperature for 30 min, and then serum-free DMEM LG was added and the matrix was detached from the well walls to allow contraction. Matrix area was measured 24 h post-detachment. To calculate contractile activity of fibroblasts the following steps were performed: (a) Measure the size of the collagen disk 24 h post-detachment in relative units; (b) measure the size of the well in the similar picture (equal to the disk size at 0 h post-detachment) in relative units; (c) calculate the percent of matrix contraction: (area of the collagen disk 24 h post-detachment)/(area of well) × 100%; (d) calculate the contractile activity of fibroblasts: 1 − percentage of matrix contraction.

### 2.7. Western Blotting

HDFs or EVs were lysed in Laemlli buffer. The protein in the lysate was quantified using the BCA protein assay, electrophoresed in SDS-PAGE gel and transferred to PVDF membrane. The membrane was incubated with primary antibodies to a-SMA (ab32575, Abcam), collagen type I (ab34710, Abcam), Bcl2 (sc-509, Santa Cruz Biotechnology), MMP2 (ab97779, Abcam), vinculin (V4139, Sigma,), GAPDH (sc-32233, Santa Cruz Biotechnology), b-actin (4970s, Cell Signaling Technology, Leiden, The Netherlands), or exosome markers (HSP70, CD81, CD63, CD9; EXOAB-KIT-1, System Biosciences, Palo Alto, CA, USA) overnight at 4 °C. After washing in TBST, the blots were incubated with horseradish peroxidase (HRP)-labeled secondary antibodies (P-RAM Iss, P-RAQ Iss, Imtek) for 1 h. The labeled proteins were visualized with ChemiDocTM Touch imaging system (Bio-Rad Laboratories, Hercules, California, USA) using an enhanced chemiluminescence (ECL) kit (Pierce, Waltham, MA, USA).

### 2.8. mRNA Level Evaluation by Real-Time Quantitative PCR

Total RNA was isolated and purified using the RNeasy mini kit (Qiagen, Germantown, MD, USA) according to the manufacturer’s instructions. On-column DNase digestion of samples was performed. The RNA was quantified and qualified using Nanodrop spectrophotometer (Thermo Scientific). The total RNA samples with the 260/280 nm ratio of 1.9 to 2.1 were used for further analyses. Reverse transcription was performed using a MMLV RT kit (Evrogen, Moscow, Russia) using oligo-dT primers and MMLV Reverse Transcriptase (Evrogen) and cDNA was synthesized as described in the manufacturer’s protocol. Real-time RT-PCR was performed using qPCRmix-HS SYBR+LowROX kit and QuantStudio 5 Real-Time PCR System (Thermo Fisher Scientific) with initial denaturation at 95 °C for 10 min followed by 40 cycles of 95 °C for 15 s, empirically matched primer-specific temperature for 10 s and 72 °C for 15 s. Primers were the following:

Collagen type I: CCCAGCCACAAAGAGTCTACA and GTTTCCACACGTCTCGGTCA; aSMA: CAATGAGCTTCGTGTTGCCC and TCTCCAGAGTCCAGCACGAT; collagen type IV: TCTGTTGGTGGAATGGGCTT and GGAAACCCGCTATCCCTTGA; RPLP0: GCTGCTGCCCGTGCTGGTG and TGGTGCCCCTGGAGATTTTAGTGG. Expression levels of the genes were calculated relative to RPLP0 levels by the comparative ΔCT method.

### 2.9. MSC-EV RNA High-Throughput Sequencing

RNA from EVs secreted by hTERT MSCs and primary MSCs (donor number *n* = 3) was isolated using miRNeasy Mini Kit (Qiagen) according to the manufacturer’s instructions. RNA was quantified and qualified using Nanodrop spectrophotometer (Thermo Scientific) by 260/230 nm ratio. Libraries for mass parallel sequencing for the analysis of miR were constructed and analyzed (Illumina, San Diego, CA, USA). Before analyzing the RNA-seq data, the adapter sequences for Sanger/Illumina 1.9/TruSeq Small RNA were first removed using the cutadapt-1.16 program, further purification using the Trimmomatic-0.38 program, followed by quality control using the FastQC program.

The RNA-seq analysis of the miRNA array was performed using the sRNAtoolbox service (parameters: miRNAs: miRBase v22; ncRNA: (Ensembl release 91 (ncRNA); length of the seed: 20; number of allowed mismatches: 2; phred score: 20). Using the TargetScan7.2, HMDD, miR2Disease, miRwayDB databases, miR associated with the development of fibrosis and their representation in MSC-EV were analyzed. Prediction of mRNA functions was aided by the Gene Ontology (GO) and Kyoto Encyclopedia of Genes and Genomes (KEGG) databases. GO and KEGG gene clustering was performed using the David 6.8 resource. Quality control, mapping and normalization of RNA-seq data of the mRNA array was performed using the BrowserGenome 1.0 service (hg38 GENCODE 22 genome). Keyword analysis was performed using scripts using the following commands: while read line; grep; awk sed; sort (uniq). Common predicted microRNA targets were analyzed using MirNet service.

Raw data are available as PRJNA592301 (ncbi/sra).

### 2.10. microRNA Level Evaluation by Real-Time Quantitative PCR

Total RNA contained microRNA was isolated and purified using the miRNeasy Mini Kit (Qiagen, USA) according to the manufacturer’s instructions. RNA was quantified and qualified using Nanodrop spectrophotometer (Thermo Scientific) by 260/230 nm ratio. Reverse transcription was performed using miScript II RT Kit (Qiagen) as described in the manufacturer’s protocol. Real-time RT-PCR was performed using miScript SYBR Green PCR Kit (Qiagen) contained 2× QuantiTect SYBR Green PCR Master Mix and universal microRNA primer on QuantStudio 5 Real-Time PCR System (Thermo Fisher Scientific). We used specific commercially available primers (Qiagen) for hsa-miR-21, has-miR-29c, and adjusted by Primer-BLAST and OligoArchitect primers (RNU6, CGCAAGGATGACACGCAAAT, Evrogen). Expression levels of miRs were calculated relative to RNU6 levels by the comparative ΔCT method.

### 2.11. MSC-EV Transfection

MSC-EV transfection was performed using Exo-FectTM Exosome Transfection Kit (EXFT20A-1, System Biosciences). Briefly, Exo-Fect solution and stabilized miR mimics or antagomirs (miRCURY LNA miRNA mimic HSA-MIR-29c-3p (YM00470481-ADA), miScript miRNA mimic Syn-hsa-miR-21-5p (MSY0000076), miScript miRNA inhibitor anti-has-miR-21-5p (MIN 0000076), miRCURY LNA miRNA HSA-MIR-29c-3p inhibitor (YI04105460-ADA), Qiagen) were added to the concentrated MSC-EV. Corresponded commercially available scramble oligos were used as a control (miRCURY LNA miRNA mimic negative control (YM00479902-ADA, Qiagen). To detect MSC-EV uptake by target cells we transfected MSC-EV with FAM-labeled mimic (fluorescein) (miRCURY LNA miRNA mimic negative control (YM00479902-ADB), Qiagen). The obtained mixture was incubated at 37 °С for 10 min and then reaction was stopped by adding ExoQuick-TC. After further incubation at 4 °С for 30 min with the polymer the transfected EVs were centrifuged at 13,000 rpm for 10 min and dissolved in DMEM LG (Gibco) supplemented with 1% penicillin-streptomycin (Gibco) up to 5× concentration and added to fibroblasts.

Uptake of MSC-EV transfected with FAM-labeled oligonucleotides (Qiagen) by HDF labeled with PKH26 (PKH26GL, Sigma) after 48 h was confirmed using confocal microscopy (Leica TCS SP5). HDF after incubation with unlabeled MSC-EV were used as a negative control.

### 2.12. Statistical Analysis

Experimental data were expressed as means ± standard deviation (SD). The Mann–Whitney U-test was performed with Statistica 6.0 software (StatSoft, Tulsa, OK, USA), and statistical significance was accepted at *p* < 0.05.

## 3. Results

### 3.1. Components of MSC Secretome Modulate the TGFbeta-Induced Differentiation of Fibroblasts into Myofibroblasts

#### 3.1.1. Acquisition of Myofibroblasts Phenotype Is Inhibited by Fractions of MSC-CM

**Figure 1:**
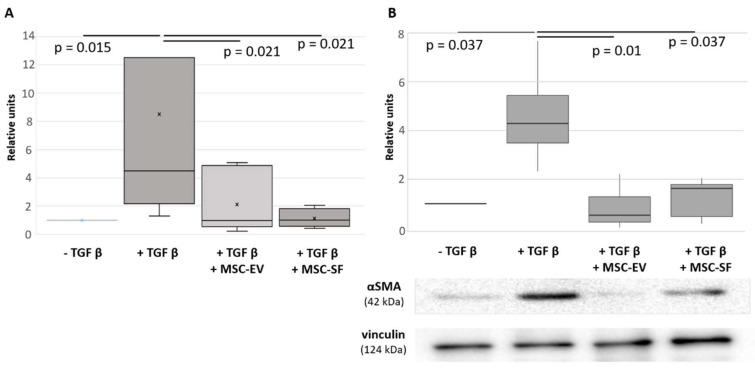
Mesenchymal stem/stromal cells conditioned medium (MSC-CM) fractions added simultaneously with transforming growth factor-beta (TGFbeta) prevented the TGFbeta-induced increase of aSMA expression in fibroblasts. Analysis of aSMA expression in serum-free cultured control fibroblasts (− TGFbeta), in fibroblasts after exposure to TGFbeta (+ TGFbeta) and TGFbeta with the components of MSC-CM (+ TGFbeta + MSC-EV; + TGFbeta + MSC-SF). (**A**) RT-PCR (*n* = 9). (**B**) Western blotting (*n* = 3).

**Figure 2:**
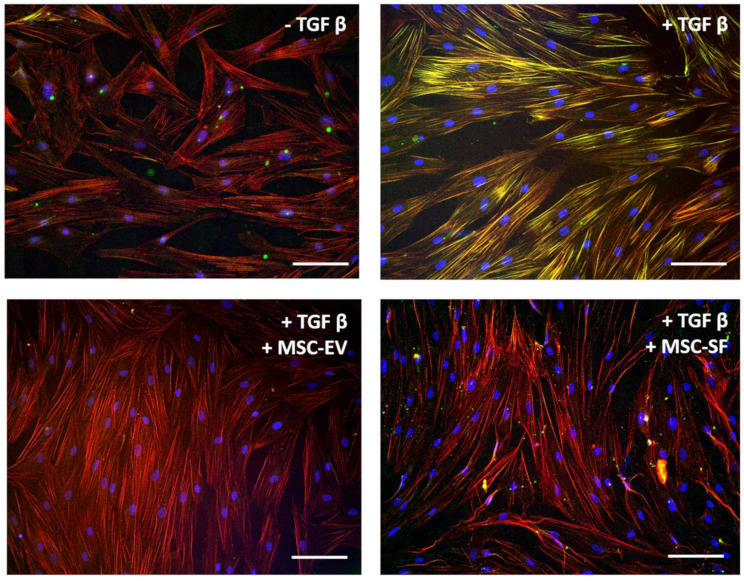
MSC-CM fractions added simultaneously with TGFbeta prevented the TGFbeta-induced aSMA redistribution into stress fibers in fibroblasts. Immunofluorescent analysis (aSMA (green), phalloidin (red), DAPI (blue)) of the content of aSMA in serum-free cultured control fibroblasts (− TGFbeta), in fibroblasts after exposure to TGFbeta (+ TGFbeta) and TGFbeta with the components of MSC-CM (+ TGFbeta + MSC-EV; + TGFbeta + MSC-SF). Scale bar: 100 μm.

To examine MSC ability to affect fibroblasts differentiation to myofibroblasts we collected MSC conditioned medium (MSC-CM) and evaluate its effects on the previously established in vitro model of TGFbeta-induced differentiation of HDF in serum-free conditions. As shown before, TGFbeta alone caused the elevation of aSMA synthesis and its incorporation into microfilaments in HDF, therefore promoting the acquisition of myofibroblast phenotype by these cells (Figure 1 and Figure 2). MSC-CM added simultaneously with TGFbeta could prevent these changes to some extent. However, the observed effects were volatile and variable. Then we separated bioactive molecules produced by MSCs into two fractions: enriched in extracellular vesicles (MSC-EV) or EV-depleted and enriched in soluble factors (MSC-SF). These fractions were applied separately on cultured HDF cultured in the presence of TGFbeta in serum-free conditions. Simultaneous application of MSC-CM fractions prevented an increase of aSMA mRNA in HDF caused by TGFbeta treatment (Figure 1A). Moreover, the application of MSC-EV fraction led to significant decrease of aSMA mRNA below the level observed in untreated cells (0.6 ± 0.05, *р* < 0.05). Consistently, the treatment of HDF with TGFbeta enhanced aSMA protein level by more than 2-fold (2.9 ± 0.1 vs. 1.13 ± 0.03, *р* < 0.05), whereas the addition of either MSC-SF or MSC-EV caused a dramatic decrease in aSMA protein level (0.4 ± 0.13 and 0.25 ± 0.15 vs. 1.13 ± 0.03, *р* < 0.05) below the level observed in untreated cells. We obtained the similar effects if normalized aSMA level to the different house-keeping controls routinely used for Western blotting (Figure S1).

Simultaneously, the application of MSC-CM fractions prevented morphological changes of HDF caused by TGFbeta. Thus, TGFbeta promoted a change the incorporation of aSMA into stress fibers. Simultaneous addition of MSC-CM fractions either prevented aSMA redistribution into stress fibers (Figure 2).

**Figure 3:**
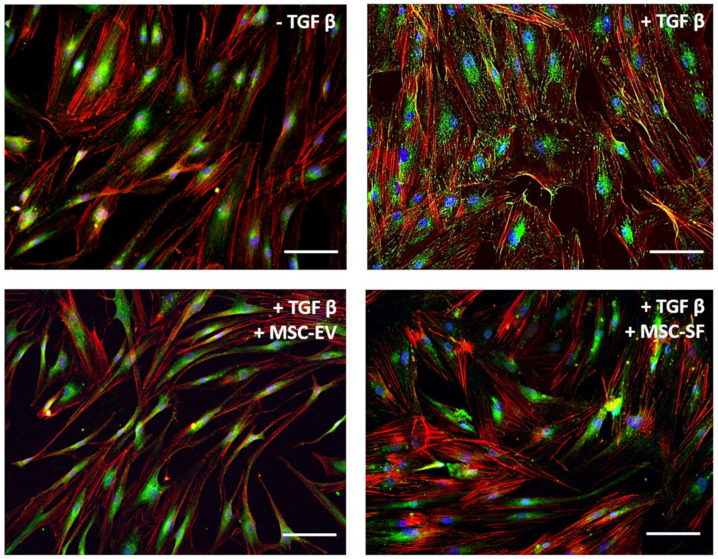
MSC-CM fractions added simultaneously with TGFbeta prevented the TGFbeta-induced intracellular redistribution of vinculin from perinuclear zone to the focal adhesion contact sites in fibroblasts. Immunofluorescent analysis (vinculin (green), phalloidin (red), DAPI (blue)) of the content of vinculin in serum-free cultured control fibroblasts (− TGFbeta), in fibroblasts after exposure to TGFbeta (+ TGFbeta) and TGFbeta with the components of MSC-CM (+ TGFbeta + MSC-EV; + TGFbeta + MSC-SF). Scale bar: 100 μm.

Since a large number of focal adhesion contacts also indicated contractile phenotype of myofibroblasts, we examined the effect of factors produced by MSC on the intracellular distribution of focal contact marker protein vinculin. TGFbeta stimulated a partial intracellular redistribution of vinculin from perinuclear zone to the focal adhesion contact sites. In cells that were simultaneously treated by TGFbeta and MSC-CM fractions, vinculin remained in the perinuclear zone as in untreated cells (Figure 3).

Taken together, these data indicate that MSC-CM fractions, both enriched in extracellular vesicles and soluble factors, prevent the acquisition of myofibroblast morphological features by fibroblasts induced by TGFbeta.

#### 3.1.2. MSC-CM Fractions Suppress Contractile Activity of Myofibroblasts

**Figure 4:**
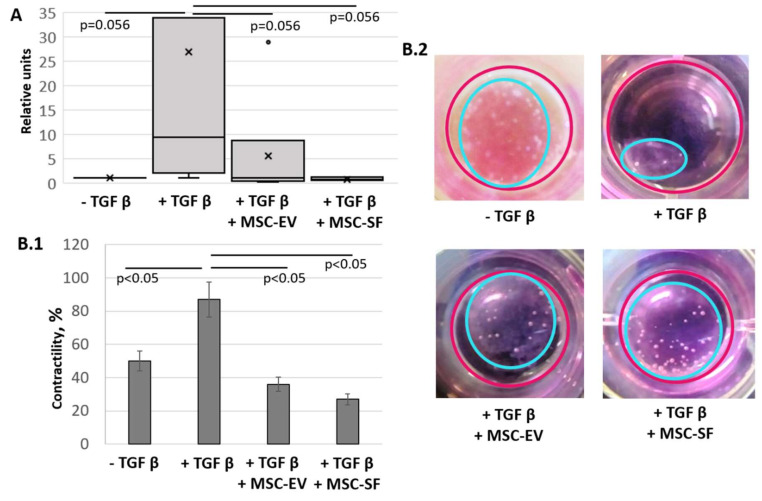
MSC-CM fractions added simultaneously with TGFbeta prevented the TGFbeta-induced increase of the collagen type I production and contractile activity of treated fibroblasts. Changes in the functional activity of serum-free cultured control fibroblasts (− TGFbeta), fibroblasts after exposure to TGFbeta (+ TGFbeta) and TGFbeta with the components of MSC-CM (+ TGFbeta + MSC-EV; + TGFbeta + MSC-SF). (**A**) RT-PCR on collagen type I (*n* = 9). (**B**) Collagen contraction assay (*n* = 3) B.1 Graph. B.2 Macro photos.

To test the hypothesis that the factors contained in MSC-CM may influence the differentiation of fibroblasts, we used in vitro tests that demonstrate the functional activity of myofibroblasts, notably the ability to overproduce the components of the ECM and the ability to ECM contraction. Therefore, we analyzed the effects of MSC secretome on the expression of type I collagen by myofibroblasts. We showed that the addition of MSC-EV as well as MSC-SF decreased the content of collagen type I mRNA (Figure 4A). Analysis by qRT-PCR showed a marked decrease in the expression of collagen type I under MSC-CM treatment compared with myofibroblast-like cells.

We also evaluated the effect of MSC-CM fractions on the ability of myofibroblasts to contract collagen gel (Figure 4B). In this case, myofibroblasts, the differentiation of which was induced by the addition of TGFbeta, reduced the area of collagen gel by more than four-fold compared with the control cells (87.7 ± 1.4% vs. 48.3 ± 14.6%, *p* < 0.05). In contrast, cells that were concurrently supplemented with TGFbeta and MSC-CM fractions had a reduced ability to cause the collagen gel to contract (37.1 ± 7.7% for EVs and 23.1 ± 4.1% for SFs, *p* < 0.05). Thus, the results indicate that MSC secretome components are able to suppress the functional activity of myofibroblasts.

**Figure 5:**
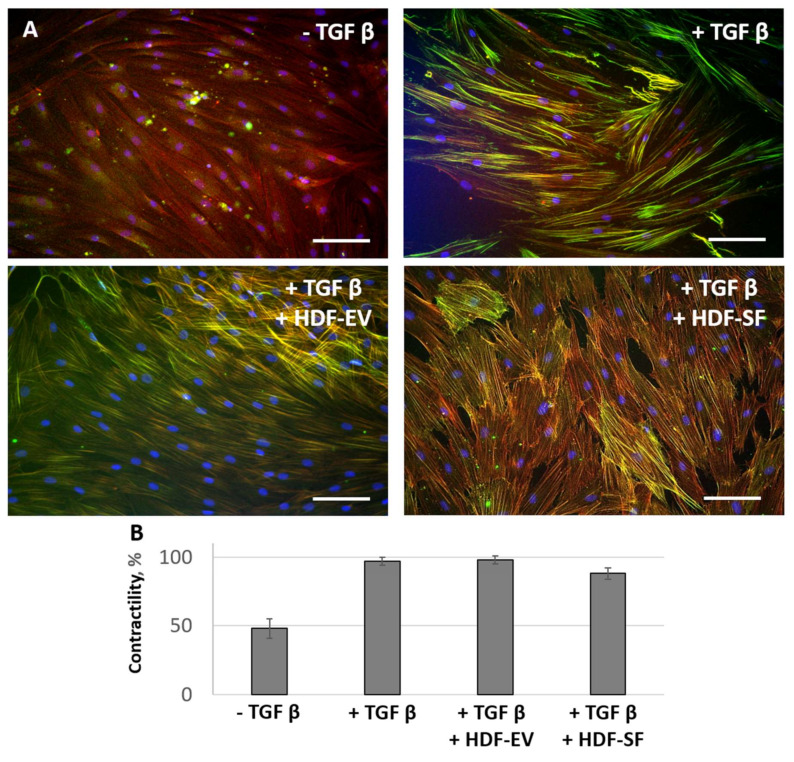
Human dermal fibroblasts conditioned medium (HDF-CM) fractions added simultaneously with TGFbeta were not able to prevent the TGFbeta-induced aSMA redistribution into stress fibers and contractile activity of fibroblasts. The expression of aSMA in serum-free cultured control fibroblasts (− TGFbeta), in fibroblasts after exposure to TGFbeta (+ TGFbeta), and TGFbeta with the components of HDF-CM (+ TGFbeta + HDF-EV; + TGFbeta + HDF-SF). (**A**) Immunofluorescent analysis (aSMA (green), phalloidin (red), DAPI (blue)). Scale bar: 100 μm. (**B**) Collagen contraction assay.

Similar antifibrotic effects were confirmed for EVs released by MSC from healthy donors (Figure S2). To confirm that the observed effects were specific to MSC, we used the condition medium from fibroblasts (as an example of distinct stromal cells). Factors produced by HDF were unable to prevent TGFbeta induced differentiation of fibroblasts to myofibroblasts as shown by aSMA distribution and myofibroblasts contractile activity (Figure 5).

#### 3.1.3. MSC-CM Fractions induce the Re-Differentiation of Myofibroblasts

Reduction in the area of fibrosis tissue can occur due to apoptosis of myofibroblasts [17] or due to reverse differentiation (redifferentiation) of myofibroblasts into fibroblasts [18]. We suggest that MSC secretome can prevent the differentiation of fibroblasts into myofibroblasts and promote the redifferentiation of myofibroblasts into fibroblasts.

**Figure 6:**
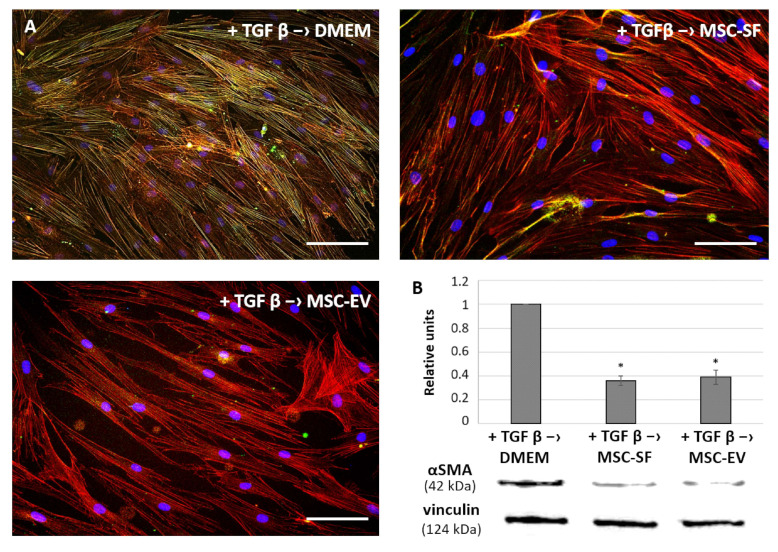
MSC-CM fractions added after the TGFbeta treatment reversed the TGFbeta-induced increase of aSMA expression and its redistribution into stress fibers in fibroblasts. Analysis of aSMA expression in the fibroblasts after exposure to TGFbeta (+ TGFbeta) and the subsequent replacement of growth medium by the components of MSC-CM (+ TGFbeta –> MSC-EV; + TGFbeta –> MSC-SF) or control serum-free DMEM (+ TGF b –> DMEM). (**A**) Immunofluorescence analysis (aSMA (green), phalloidin (red), DAPI (blue)). Scale bar: 100 μm. (**B**) Western blotting. * *p* < 0.05 (compared to TGFbeta –> DMEM group).

We induced the differentiation of fibroblasts into myofibroblasts by incubation in the presence of TGFbeta for 4 days in serum-free conditions. At the end of the incubation, the medium was replaced by serum-free DMEM or MSC-CM fractions (MSC-SF or MSC-EV). Using immunoblotting and immunofluorescence assay, we found that the addition of MSC-CM fractions to myofibroblasts caused a decrease in aSMA content in the cells by more than two-fold compared with differentiated myofibroblasts (*p* < 0.05) (Figure 6).

**Figure 7:**
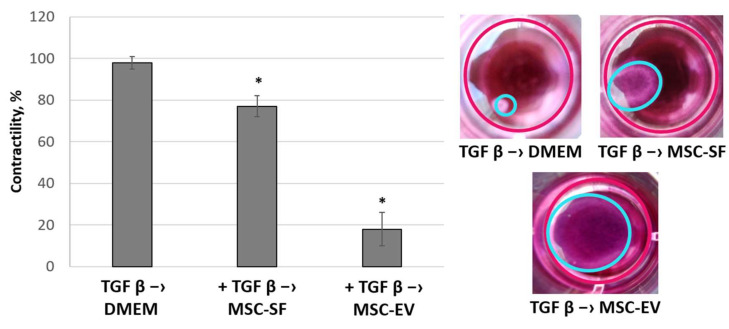
MSC-CM fractions added after the TGFbeta treatment reversed the TGFbeta-stimulated contractile activity of fibroblasts. Changes in the functional activity of the fibroblasts after exposure to TGFbeta (+ TGFbeta) and the subsequent replacement of growth medium by the components of MSC-CM (+ TGFbeta –> MSC-EV; + TGFbeta –> MSC-SF) or control serum-free DMEM (+ TGFbeta –> DMEM). Collagen contraction assay. * *p* < 0.05 (compared to TGFbeta –> DMEM group).

The addition of MSC-CM fractions also caused a decrease in the ability of myofibroblasts to reduce the collagen gel size. The most pronounced effect was exerted by the MSC-EV fraction. Myofibroblasts almost completely lost their contractile activity after MSC-EV were added (Figure 7).

Taken together, the obtained data indicate that MSC could inhibit and even reverse fibroblast differentiation into myofibroblasts induced by profibrotic agents and this effect is mainly mediated by EVs secreted by MSC.

### 3.2. RNA Sequencing Reveal miRs Associated with Fibrosis in MSC-EV

**Figure 8:**
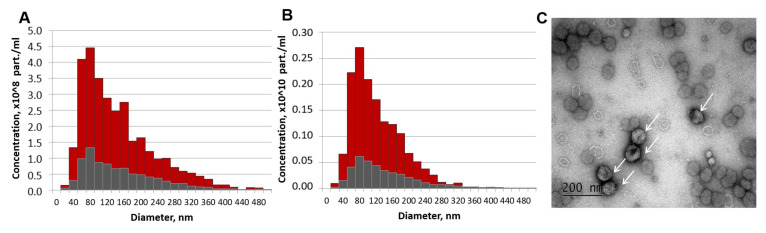
Characterization of extracellular vesicles (EVs) released from mesenchymal stromal cell (MSC) after 48 h of conditioning. (**A**,**B**) Particle size distributions of EV preparations (red) and control aliquots of MSC-CM after 30 min of conditioning (grey) for non-concentrated (**A**) and 5-fold concentrated (**B**) samples measured by nanoparticle tracking analysis (NTA). (**C**) MSC-EV imaging by the transmission electron microscopy (EVs are indicated by arrows).

EV isolated from MSC-CM were characterized in terms of morphology, particle yield, and size (Figure 8). EVs isolated with ultrafiltration demonstrated a round or cup-like shape with a mean diameter nearby 114 nm and had a particle yield of 3.12 × 10^9^ particles/mL of non-concentrated MSC-CM and 1.53 × 10^10^ particles/mL of 5-fold concentrated MSC-CM. Western blot analysis of the exosomal markers displayed that the samples were positive for typical exosomal markers, including HSP70, CD9, CD81, and CD63 (Figure S5).

**Figure 9:**
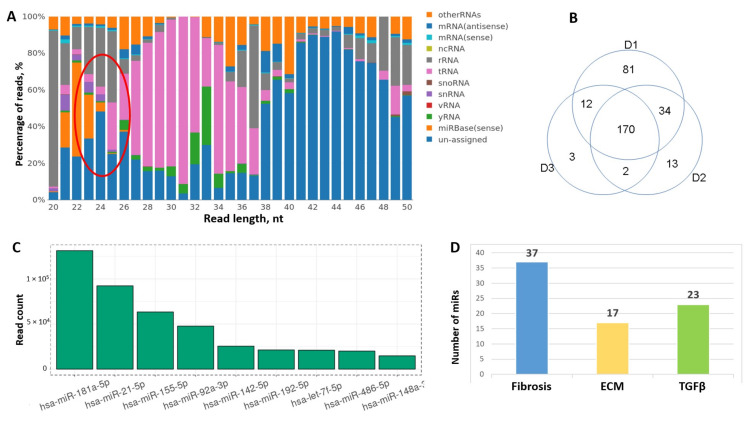
Analysis of RNA profile in MSC-EV by RNA-seq. (**A**) Different types of RNAs are represented in MSC-EV. Cluster of microRNAs marked by red oval. (**B**) Variability of microRNA representation in donor adipose tissue-derived MSC-EV by Venn diagrams. (**C**) The most abundant microRNAs in MSC-EV (top-10). (**D**) Fibrosis-associated microRNA representation in MSC-EV based on their predicted gene target analysis.

Using high-throughput RNA sequencing (RNA-seq) we found that multiple classes of RNAs are represented in hTERT-MSC-EV (Figure 9A). Using KEGG gene clustering we found that the most representative 3000 genes detected in MSC-EV included clusters associated with focal adhesion (79 genes), ECM formation (just only collagens are presented by 27 genes) and ECM-receptor interaction (39 genes), PI3K-Akt signaling pathway (90 genes), TGFbeta signaling pathway (17) and other processes involved in the regulation of fibrosis.

Among the noncoding RNAs one of the most representative and annotated class was miRs (Figure 9A). From 2652 miRs 270 were detected in hTERT-MSC-EV. Most of them were also detected in EVs secreted by MSCs derived from adipose tissue of healthy donors. However, we observed some variability between donors; thus, only 170 miRs were presented in all three biological replicates (Figure 9B). The most abundant miRs in MSC-EV are presented on Figure 9C, including miR-21, miR-155, miR-92a and others with established specific gene targets involved in the development of fibrosis. According to the results of cluster analysis it was shown that among the targets of detected miRs there were multiple fibrosis-related factors (Figure 9D). The majority of the genes substantially contributed to specific processes related to fibrosis pathways such as ECM remodeling, Wnt signaling, PDGF and TGFbeta signaling pathways. Similar results were obtained by the analysis of common targets of 170 miRs coincident between three donors of MSCs: 117 genes were related to fibrosis, 63 genes to TGFbeta signaling and 76 genes to ECM formation and remodeling. Interestingly, within the most abundant miRs in EVs about 80% were consisted between hTERT-MSCs and adipose tissue-derived MSCs from healthy donors, and almost 75% corresponded to miRs detected in bone marrow-derived MSC-EV [19].

### 3.3. MSC-EV Are Able to Transfer miRs from MSC to Fibroblasts

**Figure 10:**
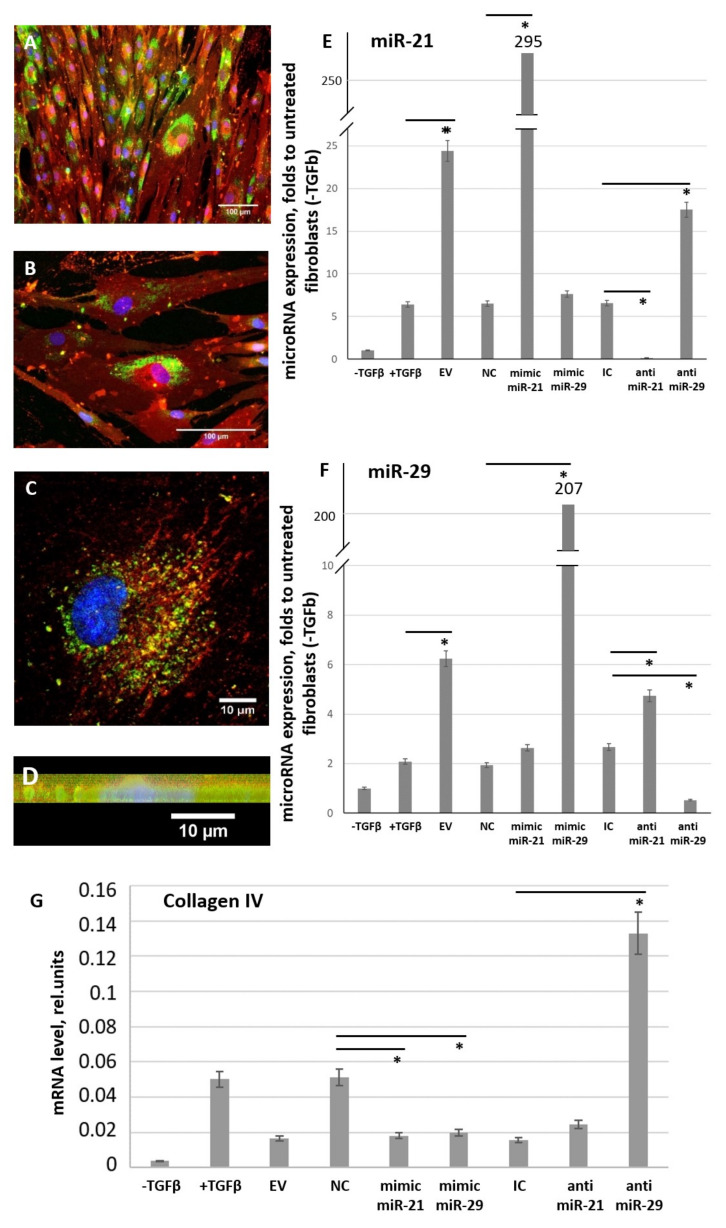
MSC-EV transfer microRNAs from MSC to fibroblasts within MSC-EV. (**A**–**D**) Uptake of MSC-EV transfected with FAM-labeled (green) oligos by fibroblasts after 48 h of incubation. (**A**,**B**) wide-field microscopy, (**C**) confocal microscopy, (**D**) confocal microscopy, sagittal section. Immunofluorescent analysis (FAM (green), PKH26 (red), DAPI (blue)). (**E**,**F**) Expression of miR-21 (**E**) and miR-29c (**F**) in cultured control fibroblasts (− TGFbeta) and in fibroblast after exposure to TGFbeta (+ TGFbeta) incubated for 96 h in the presence of MSC-EV transfected with specific microRNA mimics or antagomirs. Real-time PCR was used for the analysis. The results were normalized to the untreated control fibroblasts (− TGFbeta). (**G**) Expression of miR-21 and miR-29c target gene (collagen IV) in cultured control fibroblasts (− TGFbeta) and in fibroblast after exposure to TGFbeta (+ TGFbeta) incubated for 96 h in the presence of MSC-EV transfected with specific microRNA mimics or anti-miRs. Real-time PCR was used for the analysis. EV: MSC-EV without transfection, NC: MSC-EV transfected by negative control oligos (control for miR mimics), IC: MSC-EV transfected by inhibitory control oligos (control for anti-miRs), mimic miR-21: MSC-EV transfected by mimic miR-21, mimic miR-29: MSC-EV transfected by mimic miR-29, anti-miR-21: MSC-EV transfected by anti-miR-21, anti-miR-29: MSC-EV transfected by anti-miR-29 * *p* < 0.05 (compared to the correspondent control group).

To reveal the mechanisms of EV-mediated MSC ability to inhibit fibroblast differentiation into myofibroblasts induced by profibrotic agents we transfected MSC-EV by specific oligos mimicking selected miRs or suppressing their interaction with target mRNAs. We have chosen two overrepresented in MSC-EV miRs, one is known as profibrotic (miR-21) and another as antifibrotic miR (miR-29c). Using MSC-EV transfected with FAM-labeled oligos we confirmed the uptake of MSC-EV by fibroblasts (Figure 10A–D). Addition of MSC-EV led to the significant increase of miR-21 and miR-29c expression in recipient fibroblasts, and transfection of MSC-EV with the corresponding miR mimics dramatically enhanced (more than 10-fold) the transfer of miRs to the fibroblasts (Figure 10E,F). Transfection of MSC-EV with the corresponding antagomirs promoted significant decrease in miR-21 and miR-29c expression in fibroblasts (Figure 10E,F). Importantly, our results demonstrate that changes in miR content in MSC-EV affect the expression of other miRs in targeted cells. Thus, MSC-EV contained antagomir for miR-21 stimulated expression of miR-29c and vice versa.

The functional activity of transferred miRs was confirmed by evaluating the expression of their common gene target—collagen type IV (Figure 10G). Uptake of MSC-EV transfected with miR mimics by fibroblasts led to the inhibition of target gene expression, whereas MSC-EV transfected with miR antagomirs, especially antagomir for miR-29c, stimulate collagen type IV expression in targeted fibroblasts.

### 3.4. miR-21 and miR-29 Are Involved in MSC-EV-Mediated Antifibrotic Effects of MSC

**Figure 11:**
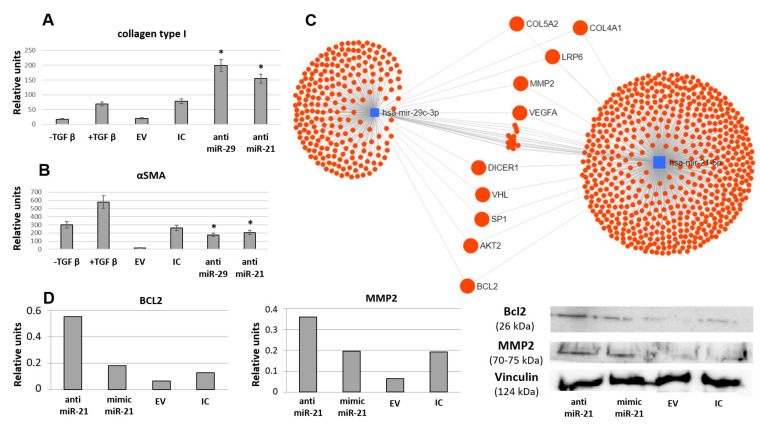
Inhibition of selected microRNAs (miR-21 and miR-29c) by antagomiRs attenuates EV-mediated MSC ability to prevent TGFbeta-induced differentiation of fibroblasts into myofibroblasts evaluated by collagen type I production (**A**) and aSMA expression (**B**), Western blotting (*n* = 3). EV: MSC-EV without transfection, IC: MSC-EV transfected by inhibitory control oligos (control for anti-miRs). anti-miR-21: MSC-EV transfected by anti-miR-21, anti-miR-29: MSC-EV transfected by anti-miR-29 * *p* < 0.05 (compared to control groups). (**C**) Common targets of miR-21 and miR-29c predicted using MirNet service. (**D**) Impact of miR-21 transferred by MSC-EV into the regulation of selected pro-fibrotic targets in fibroblasts evaluated by Western blotting (*n* = 1).

According to current evidence multiple miRs might be involved in MSC-EV-mediated regulation of fibrosis. Impact of selected miRs was evaluated using the established model of TGFbeta-induced fibroblast differentiation into myofibroblasts. Addition of MSC-EV transfected by miR mimics did not affect MSC-EV-mediated inhibition of fibroblast differentiation. However, miR-21 and miR-29c inhibitors transferred within MSC-EV into the fibroblasts increased collagen type I production and aSMA expression by these cells and thus reduced MSC-EV antifibrotic effect (Figure 11A,B). In silico analysis of miR-21 and miR-29c common targets revealed multiple important fibrosis-associated genes (Figure 11C) including ECM components (collagens IV and V, MMP2), signaling molecules (AKT kinase, Bcl2, VHL) and transcription factors (Sp1). Impact of miR-21 transferred by MSC-EV into the regulation of selected pro-fibrotic targets (Bcl2, MMP2) in fibroblasts was confirmed by transfection of EVs by miR-21 mimic or inhibitor (Figure 11D). Interestingly, both miRs could target Dicer1 which is critical for miR processing as it cleaves precursor RNA molecules to produce miRs (Figure 11C).

## 4. Discussion

Unregulated activity of myofibroblasts, highly contractile cells that deposit abundant ECM, is responsible for excessive fibrosis of different tissues. MSC located in stromal and perivascular compartments in the close proximity to fibroblasts are considered to modulate the myofibroblast activity, and in this study, we examined one of the possible mechanisms of MSC secretome effects on reparative processes, specifically by controlling the differentiation of fibroblasts into myofibroblasts.

We showed that under the influence of TGFbeta, one of the key modulators of the inflammatory response and tissue repair, fibroblasts underwent morpho-functional rearrangement and differentiated into myofibroblasts, a special “secretory-contractile type” cell [20,21]. Contrary to the expectations, MSC-CM could not effectively prevent this process. Similar results obtained by other investigators demonstrated that fibroblast growth factor-2 (FGF-2), but not the secreted factors of adipose tissue-derived stromal cells, could downmodulate TGFbeta-induced fibroblast into myofibroblast differentiation [22]. However, we demonstrated that fibroblasts treated with the MSC secretome fraction enriched with EVs or SFs showed reduced signs of differentiation, comparable with that of the negative control. The significance of these parameters in the treatment groups was substantially lower in many cases than in the negative control group. This phenomenon may indicate that during in vitro cultivation of fibroblasts the initial stages of differentiation occur and the detection of another subtype of fibroblast-like cells—proto-myofibroblasts—is initiated. These cells have an intermediate position between fibroblasts and myofibroblasts and characterized by the presence of aSMA in the fibers with a gradual increase in the level of its expression [23]. Presumably, such changes, which are uncharacteristic of fibroblasts under in vivo conditions, are associated with nonoptimal in vitro cultivation conditions. At the same time, MSC-CM fraction treatment helps to maintain fibroblasts in their “native” state. We also demonstrated for the first time the capability of MSC-EV to induce the redifferentiation of myofibroblasts into fibroblasts and specificity of MSC-CM antifibrotic effects compared to conditioned media from fibroblasts.

The EV-depleted fraction of MSC-CM enriched with SFs contains components that could affect fibroblasts, such as FGF-2 and HGF, by regulating cell proliferation and their functional characteristics [10,18,24]. Other possible mediators of MSC-SF antifibrotic effects are MMP inhibitors (like TIMP-1, TIMP-2 and others revealed in MSC secretome [9]) as well as fibrosis-related cytokines such as IL-10 and prostaglandin-E2 [5,6,7,8,9]. However, we observed that the effect of the fraction containing SFs was less stable than the effect of the fraction enriched with EVs (few negative results for MSC-SF are not shown). We assume that the procedures associated with the isolation of MSC secretome fractions might have a negative effect on the stability of SFs. At the same time, the structure of EVs makes it possible to preserve their cargo in the native state upon isolation. It should be also noted that EVs could provide more sustained and long-term effects on differentiation processes by genetic reprogramming of the targeted cells [12,25].

Currently, the contribution of EVs (exosomes and microvesicles) to the paracrine effects of MSCs is being actively studied [26,27,28]. It was assumed that the mechanism of action of EVs is associated with their ability to transfer various types of signaling molecules, including regulatory RNA, between cells [12,25,29]. There are already some studies confirming that MSC-EV can stimulate the regeneration of damaged tissue due to miRs presented in them [11,13,30]. In addition, it is important to note that the composition of miRs in MSCs themselves and in the composition of MSC-EV differs qualitatively and quantitatively [31]. This indicates the possible existence of a mechanism for sorting miRs into EVs and the high significance of a specific set of miRs in providing the paracrine effects of MSCs, particularly in the regulation of fibrosis. Thus, it was shown that EVs secreted by human umbilical cord-derived MSC could regulate the differentiation of skin fibroblasts into myofibroblasts, as well as epithelial–mesenchymal transition processes in the lungs [32]. Shentu et al. demonstrated that CD90-dependent uptake of bone marrow-derived MSC-EV by fibroblasts could block their myofibroblastic differentiation [33]. These data are in line with our results.

To reveal the possible mechanisms of MSC-EV antifibrotic effects we evaluated the pattern of noncoding RNAs in MSC-EV by RNA-seq analysis and detected many fibrosis-associated miRs coincident between EVs secreted by hTERT-MSCs or adipose tissue-derived MSC from health donors. It should be emphasized that according to our data EVs from both sources have similar antifibrotic effects and miR patterns indicating the translating perspective of using MSC-EV administration as an approach for fibrotic disease preventing/treatment. Using EVs transfection by selected miR mimics, including FAM labeled, we confirmed a direct transfer of miRs within EVs from MSC to fibroblasts. According to our data miR-21 and miR-29 are among the most represented in the cells as well as MSC-EV and at the same time involved in the regulation of fibrosis. In a study by Baglio et al. (2015) data confirming the high representation of miR-21 and miR-29 in exosomes secreted by MSCs of adipose tissue was provided [31]. It was shown that with the development of fibrosis, e.g., in the lung tissue, miR-21 expression increased [34] and miR-29 expression decreased [35]. For miR-21 predominantly profibrotic properties have been described: It inhibits the expression of SMAD7 and is involved in the activation of myofibroblast differentiation of pulmonary fibroblasts [34,36]. miR-29, on the contrary, is considered to be a representative of antifibrogenic miRs due to suppression of expression of a whole cluster of extracellular matrix proteins and factors involved in its pathological modeling in pulmonary fibrosis [37,38].

We demonstrated that addition of MSC-EV led to the significant increase of miR-21 and miR-29c expression in recipient fibroblasts, and transfection of MSC-EV with the corresponding miR mimics dramatically enhanced (more than 10-fold) the transfer of miRs to the fibroblasts. The functional activity of transferred miRs was confirmed by evaluating the expression of their common gene target (collagen type IV) in fibroblasts after the treatment by MSC-EV transfected with mimics or antagomirs compared to the corresponded control samples. However, inhibition of miR-21 in MSC-EV did not significantly restore level of collagen type IV expression, which could be explained by possible co-suppression of this target by other miRs (like from miR-29 family) with enhanced expression after the incubation in MSC-EV transfected with miR-21 antagomir.

The impact of miR-21 and miR-29c into MSC-EV-mediated antifibrotic effects was evaluated using EVs transfection with specific antagomirs. Surprisingly, both miR-21 and miR-29c inhibition attenuated MSC-EV ability to inhibit fibroblasts differentiation. It should be also noted that miR-21 considered as profibrotic is one of the most represented miRs in MSC-EV. However, many predicted targets for miR-21 are described, and some of them (i.e., PTEN as a key regulator of PI3K/AKT signaling or others) would provide opposite effects on recipient cells [39]. Some data obtained by other investigators support the anti-fibrotic effects of miR-21 observed in our study [32,33]. Moreover, analysis of common predicted molecular targets for miR-21 and miR-29c revealed many genes related to profibrotic activity. Thus, collagen IV and collagen V are important components of ECM; an increase of their expression is associated with both early and advanced tissue fibrosis. Collagen V co-assembles with collagen I into heterotypic fibrils in the cornea and skin dermis, acting as a dominant regulator of collagen fibrillogenesis [40]. It was shown that MMP2 activity is critical for TGFbeta-induced matrix contraction [41]. WNT co-receptors low-density lipoprotein receptor-related proteins 6 (LRP-6), which also interacts closely with PDGF receptor beta and TGFbeta receptor 1 at the cell membrane, involved in several processes required for myofibroblast transition [42]. Another signaling pathway, PI3K/AKT, regulates fibroblast proliferation and migration as well as collagen I production [43]. Activation of fibroblasts is also linked to the B-cell lymphoma 2 (Bcl2) family, which is involved in the induction of apoptosis, and Bcl2 antagonists partially prevent fibrogenesis in different fibrosis models [44], whereas its overexpression in fibroblasts promotes persistent fibrosis in the pulmonary fibrosis model [45]. Suppression of von Hippel-Lindau protein (VHL) in fibroblasts could protect them from profibrotic agents [46]. Such common target for both miRs as transcription factor Sp1 mediates the expression of a variety of fibrotic genes expression and its inhibition efficiently blocks ECM gene expression in vitro and in vivo [47,48]. We confirmed the impact of miR-21 transferred by MSC-EV into the regulation of selected pro-fibrotic targets (Bcl2, MMP2) in fibroblasts which indicated that in these specific conditions miR-21 within MSC-EV could have antifibrotic effects on fibroblast differentiation.

We also revealed that Dicer1 is a target for both miRs predicting possible effects of MSC-EV on miR processing in surrounding fibroblasts. Importantly, it was shown that suppression of Dicer1 expression in the myofibroblast-rich fibroblastic focus core mediated ECM sustained fibrosis progression in idiopathic pulmonary fibrosis [49]. Therefore, coordinated changes of fibrosis-associated factor expression in fibroblasts targeted by MSC-EV could explain MSC antifibrotic activity. Our results also indicated the possible interaction between miR-21 and miR-29c as MSC-EV contained antagomir for miR-21 stimulated expression of miR-29c and vice versa. However, target-specific molecular approaches should be further used to reveal the functional targets of miR-21 and miR-29c transferred by MSC-EV to fibroblasts.

It should be emphasized that the involvement of the demonstrated mechanisms in MSC regulatory functions in vivo still remains an open question. MSCs residing in the tissue stroma and perivascular niche could contribute to the myofibroblast differentiation and generation of ECM themselves, and their impact as well as interaction with resident fibroblasts is tissue-specific [1,2]. Further studies of these complicated processes using relevant animal models will be necessary to elucidate the more complex mechanisms underlying the contribution of MSC secretome components to the regulation of fibrosis in different tissues.

## 5. Conclusions

Taken together, we have shown that components of MSC secretome, especially EV-enriched fraction, could modulate the differentiation of fibroblasts into myofibroblasts and even reverse the fibrotic phenotype. We demonstrated a direct transfer of miRs within EVs from MSC to fibroblasts and the impact of miR-21 and miR-29 in MSC-EV mediated antifibrotic effects. Our study reported the existence of balanced RNA network in MSC-EV and provided a basis for further investigations of noncoding RNAs contribution to MSC-EV antifibrotic effects that could be promising for the development of novel approaches to promote fibrosis prevention and healing using cell-free therapeutic strategies.

## Supplementary Materials

The following are available online at https://www.mdpi.com/2073-4409/9/5/1272/s1, Figure S1: Analysis of aSMA expression in cultured control fibroblasts (− TGFbeta), in fibroblasts after exposure to TGFbeta (+ TGFbeta) or TGFbeta with the components of MSC-CM (+ TGFbeta + EV; + TGFbeta + SF) evaluated by Western blotting using different house-keeping controls (vinculin, GAPDH or beta-actin). Figure S2. Immunofluorescent analysis (aSMA (green), phalloidin (red), DAPI (blue)) of the content of aSMA in cultured control fibroblasts (− TGFbeta), fibroblasts after exposure to TGFbeta (+ TGFbeta) and TGFbeta with EVs released from hTERT-MSC (+ TGFbeta + hTERT-MSC-EV) or MSC from healthy donors (+ TGFbeta + MSC-EV). Figure S3. The expression of aSMA in cultured control fibroblasts (− TGFbeta), in fibroblasts after exposure to TGFbeta (+ TGFbeta), and TGFbeta with the components of HDF-CM (+ TGFbeta + EV; + TGFbeta + SF). Figure S4. Analysis of the expression of the aSMA in fibroblasts after exposure to TGFbeta (+ TGFbeta) and replacement of growth medium to the components of MSC-CM (+ TGF b –> + MSC-EV; + TGFbeta –> + MSC-SF) or DMEM (+ TGF b –> DMEM). Figure S5. Characterization of EVs released from MSC after 48 hours of conditioning.
