# Supplementary material for: Secretome of Mesenchymal Stromal Cells Prevents Myofibroblasts Differentiation by Transferring Fibrosis-Associated microRNAs within Extracellular Vesicles
Source: Cells. 2020 May 20;9(5):1272. doi: 10.3390/cells9051272 (PMC7290371; doi:10.3390/cells9051272)
Supplement: Supplementary file 1 [file cells-09-01272-s001.pdf]

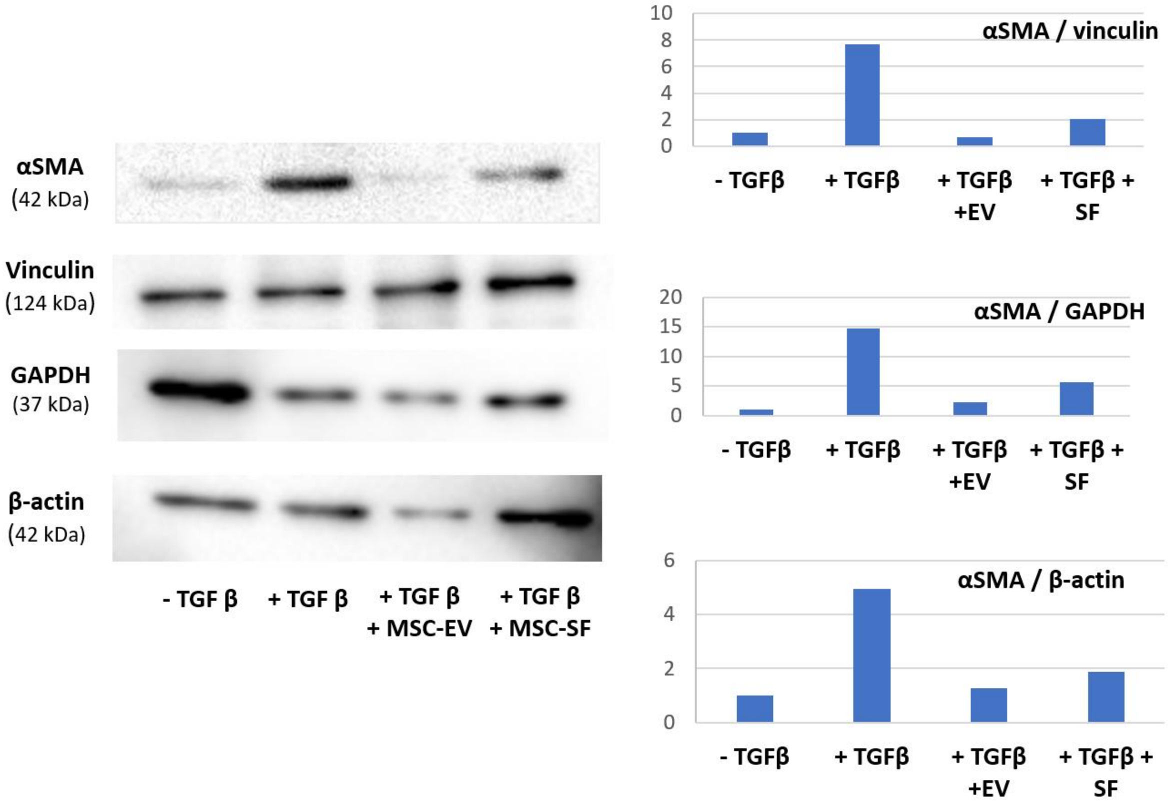

Figure S1. Analysis of aSMA expression in cultured control fibroblasts (- TGFbeta), in fibroblasts after exposure to TGFbeta (+ TGFbeta) or TGFbeta with the components of MSC-CM (+ TGFbeta + MSC- EV; + TGFbeta + MSC-SF) evaluated by Western blotting using different house-keeping controls (vinculin, GAPDH or beta-actin).

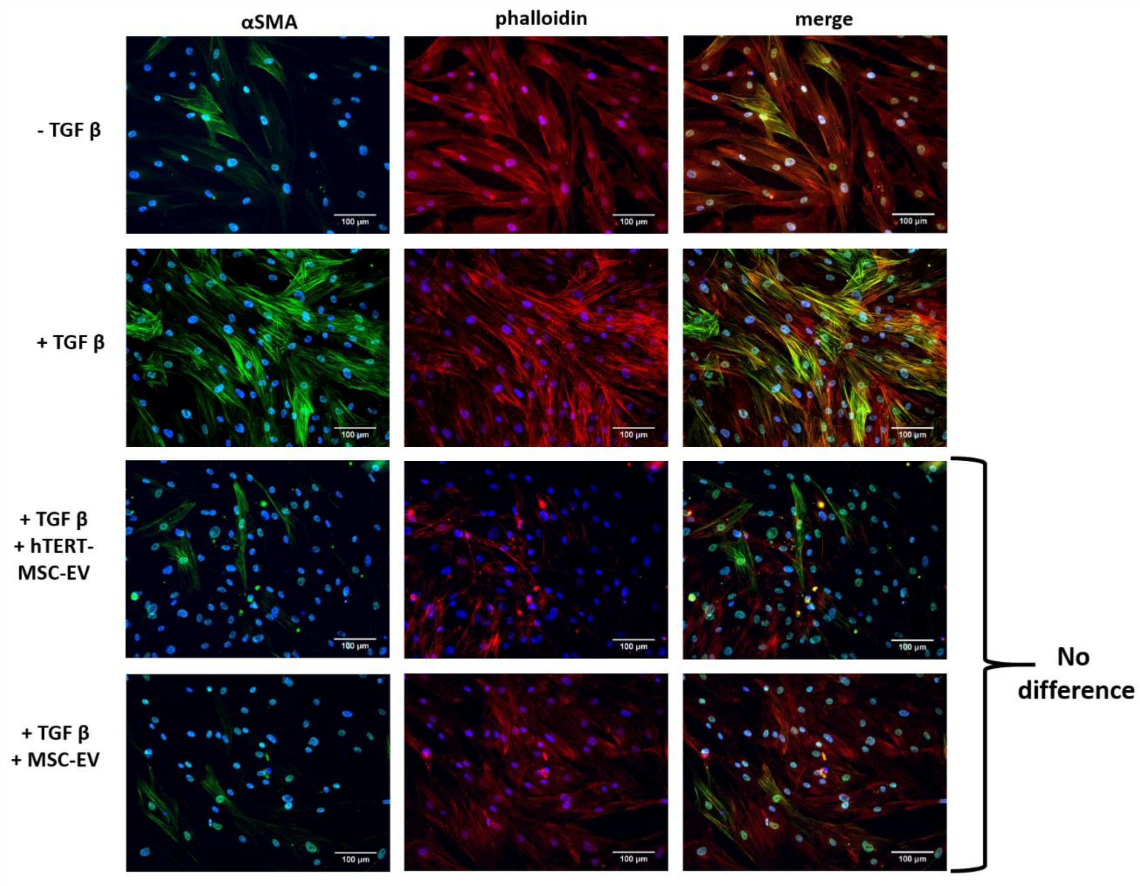

Figure S2. Immunofluorescent analysis (aSMA (green), phalloidin (red), DAPI (blue)) of the content of aSMA in cultured control fibroblasts (- TGFbeta), fibroblasts after exposure to TGFbeta (+ TGFbeta) and TGFbeta with EVs released from hTERT-MSC (+ TGFbeta + hTERT-MSC-EV) or MSC from healthy donors (+ TGFbeta + MSC-EV). Scale bar – 100 µm.

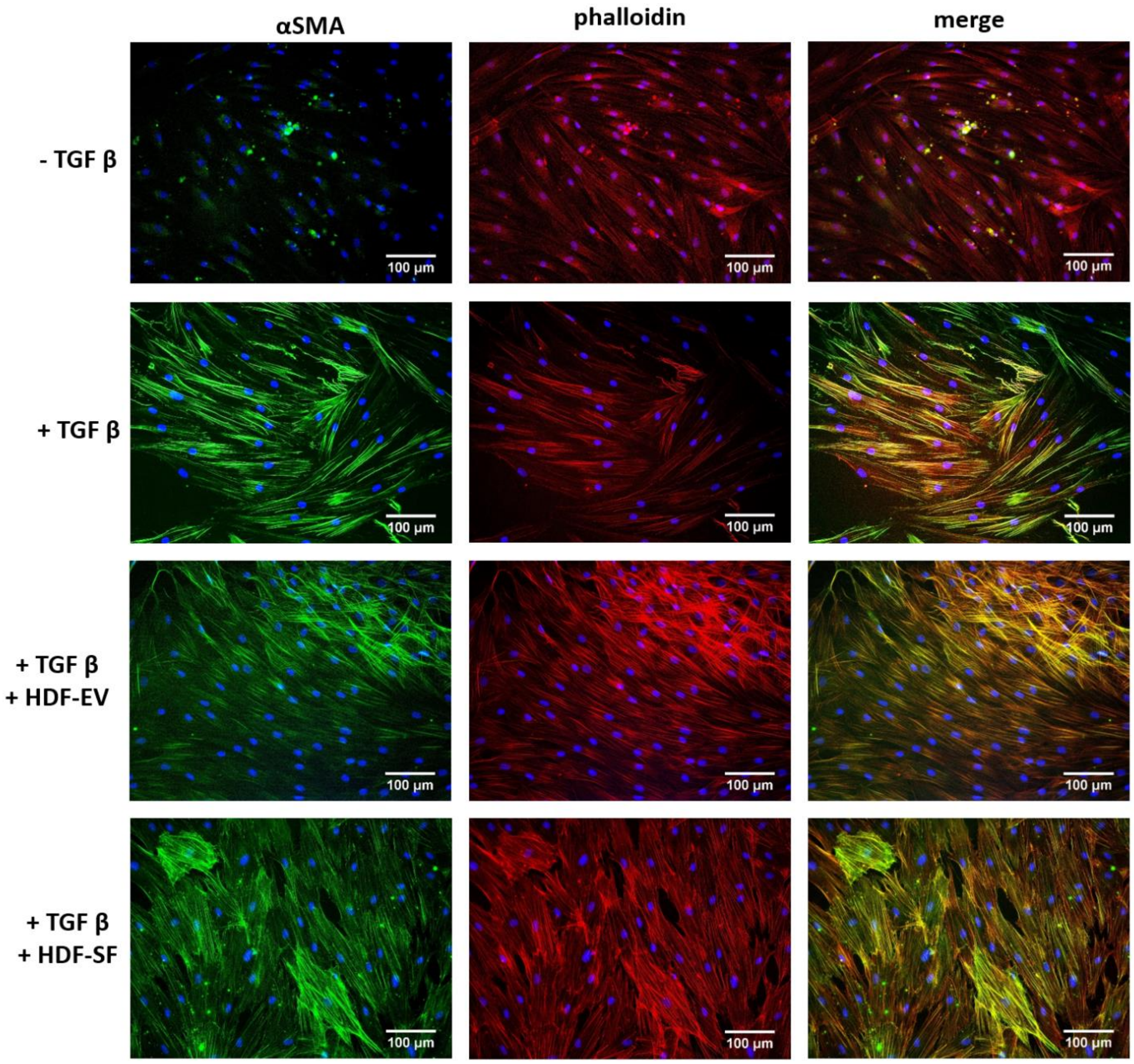

Figure S3. The expression of aSMA in cultured control fibroblasts (- TGFbeta), in fibroblasts after exposure to TGFbeta (+ TGFbeta), and TGFbeta with the components of HDF-CM (+ TGFbeta + HDF-EV; + TGFbeta + HDF-SF). Immunofluorescent analysis (aSMA (green), phalloidin (red), DAPI (blue)). Scale bar – 100 m.

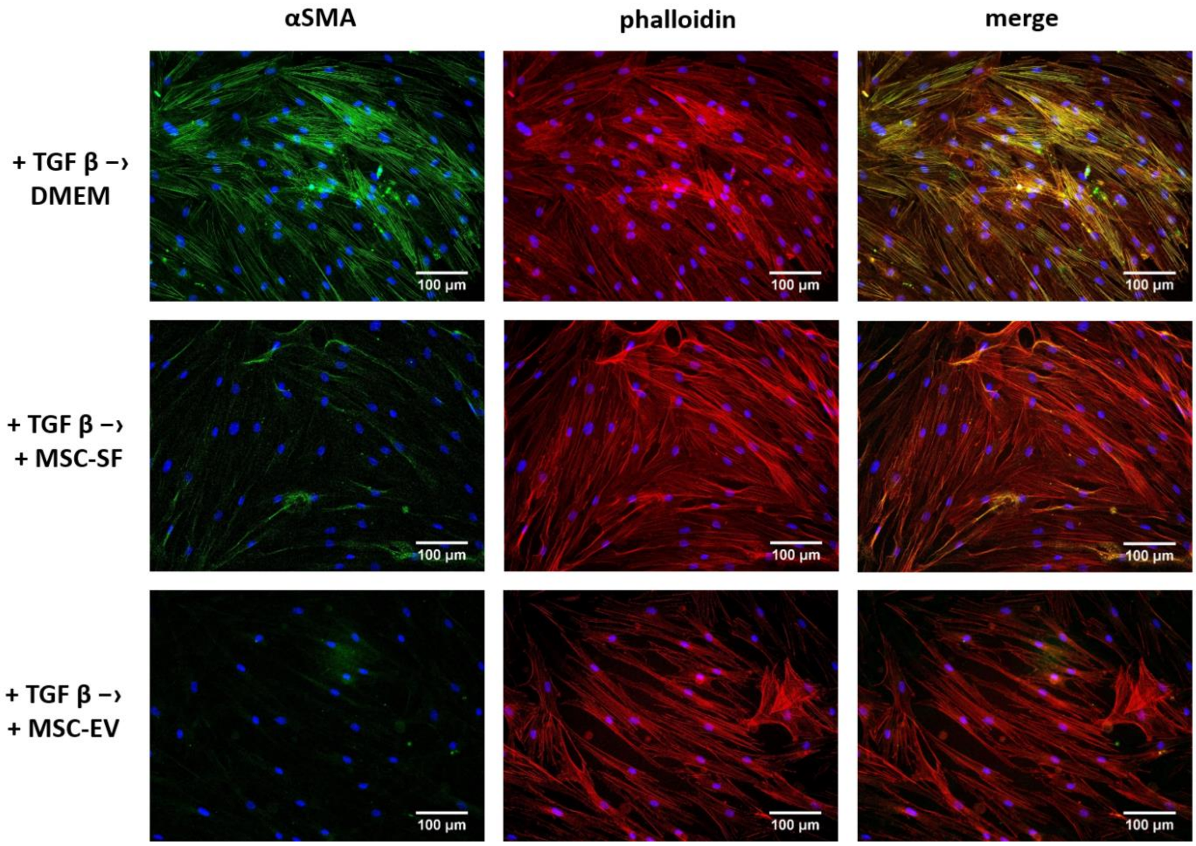

Figure S4. Analysis of the expression of the aSMA in fibroblasts after exposure to TGFbeta (+ TGFbeta) and replacement of growth medium to the components of MSC-CM (+ TGFbeta –› + MSC-EV; + TGFbeta –› + MSC-SF) or DMEM (+ TGFbeta –› DMEM). Immunofluorescence analysis (aSMA (green), phalloidin (red), DAPI (blue)). Scale bar – 100 µm.

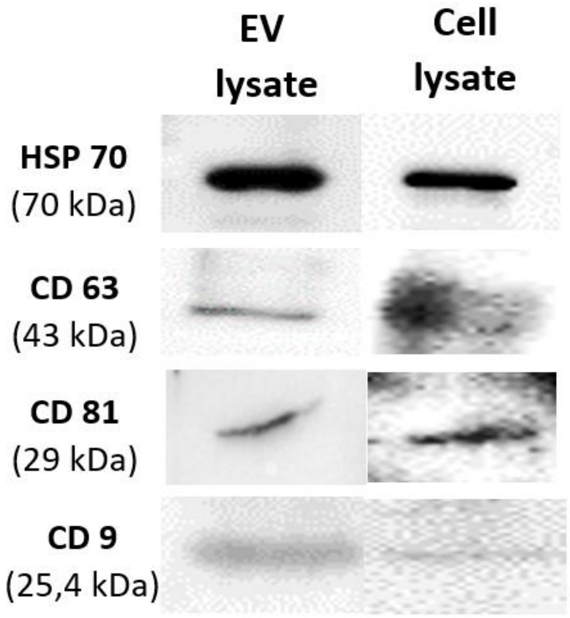

Figure S5. Characterization of EVs released from MSC after 48 hours of conditioning. EV protein markers evaluated by Western blotting in EVs and MSCs.
